# Fractional Flow Reserve-Based Patient Risk Classification

**DOI:** 10.3390/diagnostics13213349

**Published:** 2023-10-31

**Authors:** Marijana Stanojević Pirković, Ognjen Pavić, Filip Filipović, Igor Saveljić, Tijana Geroski, Themis Exarchos, Nenad Filipović

**Affiliations:** 1 Faculty of Medical Sciences, University of Kragujevac, 34000 Kragujevac, Serbia; marijanas14@gmail.com; 2 Institute for Information Technologies, University of Kragujevac, 34000 Kragujevac, Serbia; opavic@kg.ac.rs (O.P.); isaveljic@kg.ac.rs (I.S.); 3 Bioengineering Research and Development Center (BioIRC), 34000 Kragujevac, Serbia; filipovicfilip1999@gmail.com (F.F.); tijanas@kg.ac.rs (T.G.); 4 Faculty of Engineering, University of Kragujevac, 34000 Kragujevac, Serbia; 5 Department of Informatics, Ionian University, 49100 Corfu, Greece; themis.exarchos@gmail.com

**Keywords:** cardiovascular diseases, acute myocardial infarction, fractional flow reserve, machine learning, ensemble, random forest, 3D reconstruction

## Abstract

Cardiovascular diseases (CVDs) are a leading cause of death. If not treated in a timely manner, cardiovascular diseases can cause a plethora of major life complications that can include disability and a loss of the ability to work. Globally, acute myocardial infarction (AMI) is responsible for about 3 million deaths a year. The development of strategies for prevention, but also the early detection of cardiovascular risks, is of great importance. The fractional flow reserve (FFR) is a measurement used for an assessment of the severity of coronary artery stenosis. The goal of this research was to develop a technique that can be used for patient fractional flow reserve evaluation, as well as for the assessment of the risk of death via gathered demographic and clinical data. A classification ensemble model was built using the random forest machine learning algorithm for the purposes of risk prediction. Referent patient classes were identified by the observed fractional flow reserve value, where patients with an FFR higher than 0.8 were viewed as low risk, while those with an FFR lower than 0.8 were identified as high risk. The final classification ensemble achieved a 76.21% value of estimated prediction accuracy, thus achieving a mean prediction accuracy of 74.1%, 77.3%, 78.1% and 83.6% over the models tested with 5%, 10%, 15% and 20% of the test samples, respectively. Along with the machine learning approach, a numerical approach was implemented through a 3D reconstruction of the coronary arteries for the purposes of stenosis monitoring. Even with a small number of available data points, the proposed methodology achieved satisfying results. However, these results can be improved in the future through the introduction of additional data, which will, in turn, allow for the utilization of different machine learning algorithms.

## 1. Introduction

The World Health Organization estimates that cardiovascular diseases are the leading cause of death in the world with 17.9 million fatal outcomes annually. Cardiovascular diseases are responsible for significant medical, social and economic consequences globally. They represent one of the leading causes of disability, a loss in the ability to work and premature mortality, as well as place high costs on health care systems. According to the literature data, CVDs result in 31.8% of all the reported deaths in the world, and half of these outcomes are as a result of ischemic heart diseases [1,2,3].

Acute myocardial infarction is manifested through the necrosis of the heart muscle, which occurs due to coronary artery occlusion and the insufficient oxygenation of cardiomyocytes. The prevalence of acute myocardial infarction is about three million people, with more than a million deaths per year occurring in the United States [4]. Considering the serious consequences of this disease, there is a need to develop strategies for the prevention and early detection of cardiovascular risks, as well as for the rapid diagnosis of AMI for the timely application of adequate therapy [5].

The rupture of unstable atherosclerotic plaque, thrombosis and the acute reduction in blood flow through the coronary artery, with its consequent occlusion, are some of the possible mechanisms of AMI development [6,7]. A study suggested that that the characterization of culprit lesions by optical coherence tomography supported the concept that plaque erosion is more common in cases of non-ST-segment-elevation myocardial infarction (NSTEMI), while plaque rupture is more prominent in cases of ST-segment-elevation myocardial infarction (STEMI) [8].

Dyslipidemia is one of the key proposed factors in the progression of atherosclerosis. It has also been shown that a decrease in the concentration of low-density lipoprotein cholesterol (LDL-C) in high-risk patients is one of the key strategies in the prevention of ischemic heart disease [9,10]. Namely, the reduction in LDL concentration by 1 mmol/L over five years in middle-aged people reduces the risk of developing CVD by 20% [11].

The laboratory measurement of cardiac biomarkers enables the rapid diagnosis and monitoring of patients with AMI, as well as the possibility of individualizing the therapy according to the characteristics and risks of the patient. A laboratory establishment of CKMB activity is used in diagnosis, the assessment of the severity of the clinical picture and in the prediction of the prognosis of AMI. This isoenzyme, due to myocardial necrosis, shows an increase in activity in the patient’s serum after 4 to 8 h from the onset of chest pain; this then reaches a maximum within 18–24 h, and then returns back to a normal value after 24–48 h [12]. According to the data from the literature, the establishing of CKMB activity together with concentrations of myoglobin, troponin I and NT-proBNP, also have a—apart from diagnostic—prognostic significance [13].

The dominant biomarkers of myocardial damage today are certainly cardiac troponins (i.e., TnI and TnT). High-sensitivity troponins entered the clinical practice guidelines and were incorporated into the universal diagnostics definition of AMI [14]. The diagnosis of AMI is established by the detection of an increase or decrease in cardiac biomarkers, especially troponin, with at least one concentration that is larger than the 99th percentile of the healthy population and at least one symptom of ischemia [15,16].

The establishment of NT-proBNP is significant in the assessment of ventricular dysfunction and myocardial ischemia. This highly specific and sensitive cardiac biomarker is also a powerful predictor of the development of heart failure and mortality after AMI [17,18].

In current clinical guidelines, the most important diagnostic/therapeutic strategy in the management of patients with confirmed AMI is the invasive coronary angiography. By performing this procedure, the disease is indicated within 24 h in patients who meet at least one of the high-risk criteria for AMI (high cardiac troponins, dynamic changes in the electrocardiogram or a Global Registry of Acute Coronary Events risk score of >10). Percutaneous coronary intervention enables the establishment of a flow through the occluded coronary artery (which is the cause of AMI), as well as helps in gaining insights into the condition of other blood vessels.

Fractional flow reserve measurement is used to quantitatively assess the severity of the coronary artery stenosis identified during invasive coronary angiography. FFR is defined as the ratio between the maximum possible blood flow in the diseased coronary artery and the theoretically possible maximum blood flow in the normal coronary artery. During angiography, FFR is measured using a wire (catheter) for measuring coronary pressure, as well as by calculating the ratio between the coronary pressure distal to the coronary artery and the pressure in the aorta when under conditions of maximum myocardial hyperemia. This ratio shows the potential decrease in the flow distal to coronary stenosis. In healthy people, the FFR is 1, whereas an FFR lower than 0.75–080 indicates myocardial ischemia. FFR values less than 0.75 indicate the need for revascularization [19,20].

Multidetector computed tomography fractional flow reserve (MDC FFR) is used for a more elaborate assessment of the hemodynamic significance of coronary artery stenosis when compared to classic FFR. This quantitative technique is built on processing, based on a mathematical model of fluid dynamics, the obtained data.

A decision tree algorithm is a supervised classification algorithm that is based on a binary tree structure. This algorithm splits the ranges of input variables to create conditions with which the dataset can be split between two or more classes. Each condition represents a node of the binary tree. Each node branches into two other nodes, where each branch represents one of the two possible outcomes of the set condition. The leaves of the decision tree represent classes, which are then assigned to the samples of data.

A random forest classification algorithm is a supervised classification algorithm that is based on an ensemble of multiple decision tree models. Each decision tree model contained within the random forest has the same aforementioned way of making decisions, but it is trained with different, randomly selected subsets of data. Each decision tree is capable of making its own decisions, but the final output of the random forest is made by counting the number of times each class was chosen by the decision trees and by selecting the class that was chosen the highest number of times. The random forest approach reduces variance in classification with its voting process when compared to a single decision tree, and it achieves this while also reducing the overfitting of the model by feeding each tree with a smaller subset of the initial set of data.

In recent times, artificial intelligence has been gaining a strong foothold in medical science. Machine learning and deep learning models are gaining a widespread use in the automation of disease classification, disease development over time, as well as risk monitoring through the use of classification and regression analysis algorithms. Several studies have been conducted for the purposes of FFR patient risk classification [21,22,23]. All of these studies used data comprising computed tomography angiography (CTA) images for training convolutional neural networks. In this paper, we propose a methodology based on an ensemble of machine learning models for the purposes of patient risk classification through fractional flow reserve measurements using demographic and clinical data. The created system is meant to serve as a decision support tool for medical experts.

## 2. Materials and Methods

**Figure 1:**
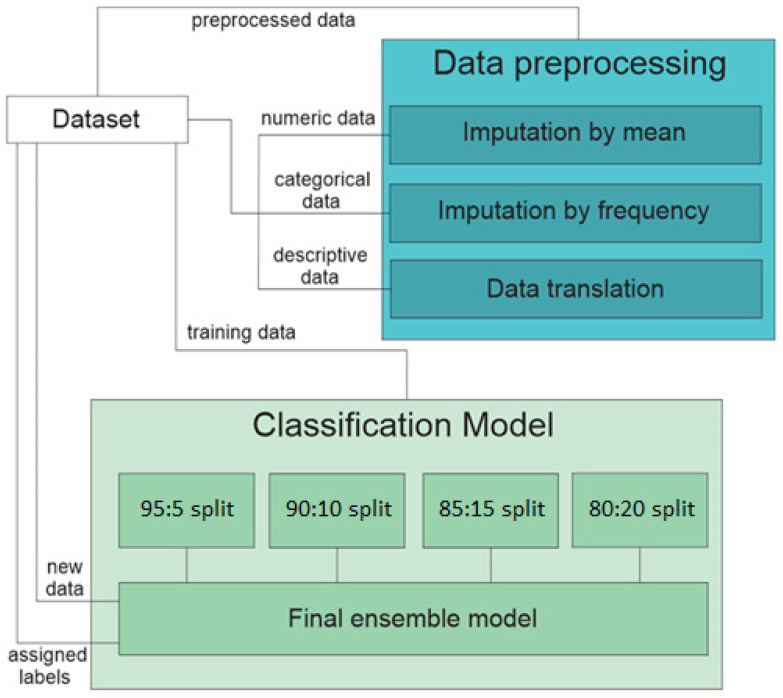
Graphical representation of the applied methodology.

This section of the paper contains information on the available data, as well as the methodology used for the data preprocessing and the creation of the final classification model. The methodology used is depicted in Figure 1.

### 2.1. Dataset

Our dataset is composed of the clinical data gathered from patients in the form of biomarkers and the descriptive data points regarding primary and follow up diagnosis, as well as the descriptive data points that define the position and degree of stenosis and lesions in three defined arteries from the left and right coronary artery trees. Along with the aforementioned data collected directly from patients, our dataset contains simulated FFR values, which represent the target to be used in the classification of patients into high-risk or low-risk classes.

During the visits, blood samples were taken from the patients according to the usual standards of clinical biochemistry. All biomarkers were determined in the Laboratory Diagnostic Service of the University Clinical Center Kragujevac. Standard laboratory methods were used in all the patients to establish the following values: hematological parameters (total number of leukocytes); concentrations of biochemical parameters (glucose, urea, creatinine, uric acid, cholesterol, triacylglycerols and LDL); the enzyme activity of cardiomyocyte damage markers (CK, CKMB, AST and LDH); and cardio-specific proteins (hs TNI and NT-proBNP). Hematological parameters were established on a DxH900 hematological counter, Beckman Coulter Analysers and biochemical parameters. The cardio-specific enzymes were established on an Oly AU 680 biochemical analyzer and on Beckman Coulter Analyzers. An Abbot Allinity immunochemical analyzer was used to establish the concentration of hsTNI, whereas the concentration of NT-proBNP was measured on a Cobas e411 immunochemical analyzer (Roche Diagnostics, Mannheim, Germany). All laboratory measurements included the implementation of regular internal and external quality controls in accordance with the recommendations of good laboratory practice. The study conduction was complied with the code of ethics of the World Medical Association (Declaration of Helsinki), and it was also approved by the Ethics Board of University Clinical Centre Kragujevac.

In our study, we have included patients suffering from coronary artery disease, where 80% had a history of AMI. Most of the patients had between 40% and 50% of stenosis, which meant that they belonged to the intermediate class of coronary artery stenosis; this was the reason virtual FFR was applied as a validation tool.

All the features used in the creation of a machine learning model, as well as their data types and ranges, are shown in Table 1.

The dataset contained data on 276 patients, of which 181 had simulated FFR values. Of the 181 labeled patients, 123 belonged to the low-risk class and 58 belonged to the high-risk class. Our approach included the training of a machine learning model with 181 patients for which the simulated FFR values were available. The geometries for the numerical simulation of FFR for these 181 patients were taken from the invasive coronary angiography images. In addition, a 3D finite element model was built based on the methodology published in [24]. Details on the 3D reconstruction and analysis are given in Section 2.3. We have already published several papers related to the numerical simulations, and we have obtained a good match with the measurements of FFR [25,26]. Now, this methodology was used as a standard to compare with the results of ML model.

The remaining 95 patients were patients who had suffered an AMI in the past, as well as had clinical and demographic data available; however, these patients were unlabeled because their geometric coronary angiography data were not available. Because the 95 unlabeled patients still represented possible real world combinations of the feature values, these patients were used for missing data imputation. However, the labels could not be assigned to those patients, so they could not have been used for the validation of machine learning models in any way. The 95 unlabeled patients were fed into the final classification model in order to demonstrate the application of the proposed methodology on those patients with unknown FFR values. The main challenge was that the data of those 181 patients was a very low amount of data that was used for training. After this, the model was applied to predict the values for the new 95 patients (for which the FFR values were unknown). Nevertheless, since the final machine learning model was meant to classify the patients using non-geometric parameters, it was expected that the model would achieve similar classification results to the results obtained during the testing on labeled data. It is important to emphasize that the application of the proposed methodology to the unlabeled data cannot be viewed as a validation attempt. The application of the proposed methodology represents a transfer of the learned medical knowledge from the labeled subset of patients to the patients for whom the ground truth was unknown. The added value of the proposed methodology was that the numerical calculations with a combination of the real measurements of the FFR could help in the future to significantly increase the size of the dataset, as well as increase the accuracy of the proposed ML models.

The problem of missing data in a dataset was tackled using a conventional approach, whereby the missing samples were filled in depending on the type of data contained in the column in question. Namely, the numeric data were replaced by the mean value of the already present values in the examined column, and the categorical data were replaced by the most common value in the column. For the purposes of data imputation, there was an attempt at using a multiple imputation approach via chained equations, but the results were very poor because of the low correlation between the different features; as such, the aforementioned approach yielded far greater results.

As for the descriptive data regarding stenosis and the lesion values of the three arteries, they were required to be translated into numeric values so that they could be used during the training of the classification model. The problem arose with the formatting of the descriptive data, and this was because very similar situations were described in completely different ways; as such, there was no way of translating these data other than translating them directly by hand and approximating the meaning. The data were translated as follows:

- Data that contained percentile values for the narrowing of the observed artery were translated as a numeric sample corresponding to the percentage value.
- Data that contained an approximation of the narrowing in the form of a range of values were translated as a numeric sample that corresponded to the average value of the observed range.
- Data that did not contain percentage values of the narrowing but did have an indication that the narrowing was not substantial were translated as if they held information of a 10% narrowing.
- Data that did not contain percentage values of the narrowing but did have an indication that the narrowing was very minor were translated as if they held information of a 5% narrowing.
- Data that did not contain percentage values of the narrowing but indicated an orderly arterial lumen were translated as if there was no narrowing at all.
- Data that did not contain any indication of the size of the narrowing nor contained the previously mentioned phrases with which the narrowing was estimated were not translated at all. Instead, they were approximated as a mean value of all of the other translated values.

Lastly, the available simulated FFR values were written in the form of a floating-point notation between the values of 0 and 1. These values had to be transcribed into categorical values that represented the risk class of the patient so that they could be used as output values of the classification model.

With regard to FFR, the patients could be divided into 3 risk classes. The low-risk class was defined by an FFR greater than 0.8, while the high-risk class was defined by an FFR lower than 0.74. There also existed a class between the values of 0.74 and 0.8, which was defined as a border class because the patients in this range could be considered both high-risk and low-risk; the final classification was the doctor’s prerogative [27]. When transcribing the data, this border class was viewed as a part of the high-risk class and was merged. This was performed because of the inherent risk of falsely putting high-risk patients as anything other than a high-risk class.

### 2.2. Data Correlation

After data preprocessing, we ran tests to find the correlations between the input values and the designated output FFR value. These correlations were calculated with the aim of expanding the dataset using high correlation features to create more labeled data. The correlation between the features and the patients’ FFR is shown in Table 2.

With the available data, it was not possible to expand the dataset because there were no features that had a high correlation with the FFR values. The classification model had to be created using only the initial data, which presented a challenge due to the small amount of labeled data.

### 2.3. 3D Reconstruction and Analysis

Three-dimensional models of the right and left coronary arteries were reconstructed from DICOM angiography images. An eight-node brick element was obtained as the final element. PAK-F software, version 2023 [28] was used for the numerical solution of the fluid flow problems. The three-dimensional flow of a viscous incompressible fluid that is considered here is governed by the Navier–Stokes equations [28], and its continuity equation can be written as follows:

(1) $$\rho \left(u_{i}\cdot \nabla \right)u_{i}+\nabla p_{i}-\mu \Delta u_{i}=0$$

(2) $$\nabla u_{i}=0$$

where *u_i_* is velocity, *p_i_* is pressure, *μ* is the dynamic viscosity and *ρ* is the density of blood. The first equation represents the balance of linear momentum, while Equation (2) expresses the incompressibility condition. By applying the Galerkin method on the previous two equations, we obtained the final form of the discretized Navier Stokes equations as follows:

(3) [1ΔtM+K^n+1vvi−1KvpKvpT0]{ΔViΔPi}={Fn+1exti−10}−[1ΔtM+Kn+1vvi−1KvpKvpT0]{Vn+1i−1Pn+1i−1}+{1ΔtMVn0}

*FFR* is defined as the ratio of the maximum flow through a coronary artery in the presence of stenosis with the maximum flow through a normal coronary artery [29]:

(4) $$FFR=\frac{Q^{S}}{Q^{N}}$$

where *Q^S^* is the flow through an artery with stenosis, and *Q^N^* is the flow through an artery without stenosis. The flow through an artery without stenosis can be calculated as follows:

(5) $$Q^{N}=\frac{p_{a}-p_{v}}{R}$$

where *p_a_* is the mean aortic pressure, *p_v_* is the mean venous pressure and *R* is the resistance through the heart. The flow through the artery with stenosis is calculated in a similar way:

(6) $$Q^{S}=\frac{p_{d}-p_{v}}{R}$$

where *p_d_* is the mean distal pressure in coronary arteries with stenosis. When we substitute Equations (6) and (5) into Equation (4), we obtain the following:

(7) $$FFR=\frac{p_{d}-p_{v}}{p_{a}-p_{v}}\approx \frac{p_{d}}{p_{a}}$$

In the case of healthy arteries, the *FFR* value is 1. Based on clinical trials, the critical value for stenting is any value that is ≤0.75.

Blood was considered as an incompressible Newtonian fluid with a dynamic viscosity of *µ* = 0.00365 Pas and a density of *ρ* = 1050 kg/m^3^. In order to calculate the numerical FFR value, two separate simulations were performed for each case. A pressure of 100 mmHg was applied at the inlet, and the flow rates of 1 and 3 mL/s were applied at the outlet. Patient-specific microvascular resistance was considered a specific Windkessel boundary condition. This was algebraically coupled to calculate the outlet pressure and flow, which was informed in each time step of the 3D computational fluid dynamics simulation [26].

### 2.4. Classification Model

The main problem encountered in the development of our classification model was the inability to test the model’s performance because of the small amount of data labeled with a risk class. More specifically, the data from 181 patients were not enough to build a comprehensive test set. To overcome this problem, we resorted to using an ensemble, which consists of a great number of less complex prediction models [30].

These less complex prediction models were also smaller ensemble models that were created using the random forest classification algorithm. First, we trained 19 random forest classification models that consisted of 50 decision trees and were without constraints in regard to the minimum samples required for creating branching nodes and leaves. The 181 labeled patients from the original dataset were split into 20 groups of data samples, each containing 5% of the data and including both high-risk and low-risk patients. Each of the models was trained using a different configuration of 19 groups of training samples, and they were tested with the one remaining group of samples. After that, we trained more models with every possible configuration of 18, 17 and 16 groups of training samples, as well as tested them with their respective combinations of 2, 3 and 4 remaining test sample groups.

The major drawback of the standard approach is that, when a model makes a wrong prediction with such a small test set, the final accuracy metric was severely impacted. To resolve this problem, we kept only the models that were deemed capable of predicting their respective test sets very precisely. In the case of models trained with a configuration of 19 training samples, only those models that predicted 6 out of 9 test samples correctly were kept. In the case of models that used bigger test sets, only those that achieved the set threshold for classification accuracy were kept. We achieved this by setting thresholds of 66%, 75%, 75% and 80% accuracy for the models being tested with 1, 2, 3 and 4 test sample groups, respectively. Only models above the given threshold were kept while the others were discarded. In the end, a total of 2785 classification models were obtained, and each model was trained with different configurations of the samples from our starting dataset.

The final model we created was an ensemble of these 2785 models. Each new sample from the original dataset was fed to every one of these models in succession. After new pieces of data were fed to all of the models in this ensemble, the final decision was made by counting up the outputs for each class and picking the one that was chosen most frequently.

## 3. Results

This section of the paper provides a review of the results acquired from the training and testing of the classification model, as well as presents the possible approaches through which to improve its performance in the future.

### 3.1. Classification Results

In the starting dataset, an imbalance can be noticed between the samples belonging to the high-risk class, of which there were 58 samples, and the low-risk class, of which there were 123 samples. Moreover, there was a risk of falsely classifying the patients into the low-risk class when they should be in the high-risk class. Hence, we first opted to evaluate our model using the F1 score metric on the high-risk class. However, we experienced some difficulties evaluating the final model in such a manner.

In the main, the F1 score metric was spoiled due to its tendency to evaluate the model through only using the results achieved from a single class. In this case specifically, there were multiple lower-level models that had been tested using only samples belonging to class 1, or, in this case, the low-risk class. In these situations, the F1 score was drastically lowered even though it was able to predict multiple test samples correctly. The problem was that the sizes of the test datasets were quite small and could not be increased in any way.

As a result, prediction accuracy was chosen as the main evaluation metric of our model’s capabilities. The final model’s accuracy was calculated as a mean of the accuracy of each of the lower-level models that were used in creating an ensemble for the final model. This accuracy metric is a simulated metric that evaluates the average performance of all the final model’s pieces instead of the entire final model. The classification model achieved an estimated prediction accuracy of 76.21%. The average performances for the models trained with different configurations of training and test sets are shown in Table 3.

### 3.2. Feature Importance

The importance of the features used during the training process of our classification model varied from one lower-level model to the next. This variation was caused by differences in the training data sample groups, which affected the model’s ability to consolidate the concrete values for the importance of certain features.

However, some features varied less than others. Every training feature at our disposal was a crucial part of at least some of the classification models, but those features that did not vary much were the best features in a majority of the classification models and served as a backbone to the final classification ensemble. In the end, feature importance was calculated for the final model as a whole, and this was expressed as a mean value of the feature importance across all of the lower-level models. These feature importance values are shown in Table 4.

The feature importance of ac. uricum, pBNP, leukocyte, troponin and glucose varied very little between the different models. The feature importance of cholesterol/HDL, AST, urea and creatinine varied heavily between the models, ranging from being extremely important in some and mostly redundant in others. The feature importance of the atherosclerosis index, trig, smoker and gender was quite low across the board; however, the number of good models was slightly reduced every time one of these features was omitted from the training process.

These feature importance values, especially those that had very little variation between models, can be used to explain the learning and decision-making process—after the evaluation of the patient’s state—of the final model to the patient.

### 3.3. Numerical Simulation Results

**Figure 2:**
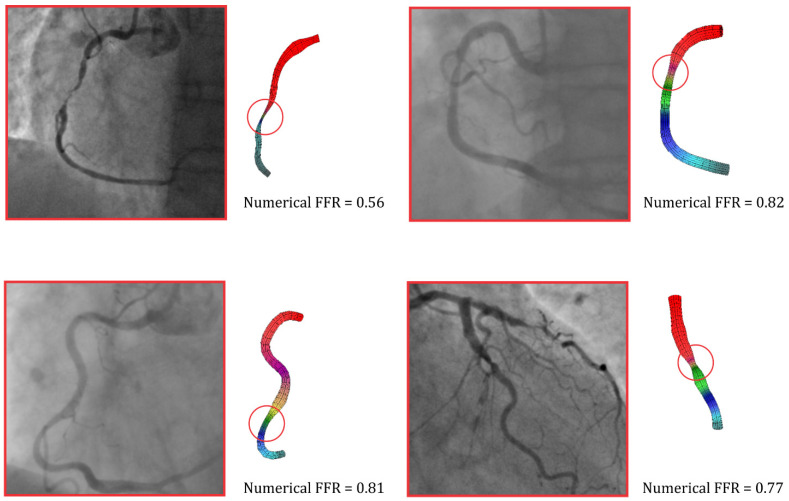
Pressure distribution, based on finite element analysis, in the coronary arteries. The FFR value was calculated based on numerical simulations.

Figure 2 shows the results of four patients after a numerical simulation in the case of a flow rate of 3 mL/s. This flow rate was a standard maximum flow for the measurement of FFR when adenosine was intravenously administrated. A red circle can be seen in Figure 2, which marks the observed stenosis on the artery. As already mentioned, a good agreement between the numerical simulations and the measurements of FFR was obtained, and this was the reason we used numerical results to validate the ML model [25,26].

## 4. Discussion

The main limiting factor during the creation of a classification model, for the purposes of classifying the patients based on their FFR, was the very low amount of available labeled data. The low amount of data severely limited the possibilities when choosing the base algorithm and tuning the parameters of the classification models. We hypothesized that the classification process could be drastically improved if there were more labeled data samples.

Also, the current model’s prediction capabilities could be improved by adopting a different approach to building the ensemble. One of the ways through which to achieve this improvement is to fine tune the models by utilizing grid search during the training process. Fine tuning would exponentially increase the training time of the model, but it would also potentially increase its prediction performance in the end. Another approach that could be utilized was the creation of different types of classification models with the same configurations of training and testing datasets [31].

Furthermore, even though the imbalance between classes was not large, this imbalance, when paired with the size of the entire labeled dataset, rendered the use of traditionally good ensemble inclusions impossible. Namely, when working with small datasets, machine learning algorithms such as K-Nearest Neighbors and the Support Vector Machine achieve good classification results. However, each of these approaches had some drawbacks when used in this particular situation.

The Support Vector Machine algorithm is a kernel-based classifier, which divides the training data using multidimensional hyperplanes, the dimensionality of which is dependent on the dimensionality defined by the model input parameters. As an algorithm, it is capable of perfectly separating a dataset based on training data samples while keeping the Euclidean distance between the physical representations of the training data points in multidimensional space at the maximum. However, problems arise with the generalization capabilities of such models for newly introduced data. For this reason, a coefficient of error tolerance was introduced, which allows the algorithm to make minor mistakes during training but also increases the potential to better generalize when making decisions in the future. The problem in this particular situation arises when any high-risk patient is present in the test set as this reduces the number of available high-risk patients for training. Any value of allowed error tolerance renders the models incapable of predicting the high-risk class in an acceptable manner.

Similarly, the K-Nearest Neighbors algorithm, while not an algorithm that creates a mathematical model in the true meaning of those concepts, is still capable of splitting a multidimensional hyperspace into sections belonging to observed classes. The class separation of this algorithm is based on the proximity of similar training data points in the n-dimensional space. The high-risk patients defined two dense clusters within the aforementioned space, and this was achieved by clearly separating the zones within which the patient would be considered as under a high risk of AMI from those that could be considered to be under a low risk of suffering AMI. Introducing high-risk samples into the test set reduced the density of these clusters. This, consequently, greatly reduces the area inside the n-dimensional hyperspace within which the patient could be classified as high-risk, or, as in some situations, where those zones would be eliminated.

With the increase in the size and diversity of the dataset that was available for model training, the inclusion of a classification model other than the random forest model in the final ensemble became a possibility. While the introduction of new models would increase the time needed for training and parameter optimization, as well as slightly increase the time needed for prediction, the introduction of these models would, on the other hand, further reduce the output variance and greatly increase the versatility of the final ensemble.

High-quality data are seldom available in large amounts in fields of research like medicine due to ethical guidelines and patient privacy protection. Furthermore, medical data that are tied to specific diseases are, in some cases, region-specific, and they are also much sparser in some locations compared to others. In order to address these challenges, the proposed methodology serves as a proof of concept for a way in which to improve the automatic diagnosis approach when using a small amount of available data.

The main limiting factor of this study was the small amount of real data available as input to the ML model. Commonly used techniques for dataset enhancement that include the generation of new data through oversampling and the estimation of labels for unlabeled samples when using multiple imputations through chained equations are not always applicable to certain datasets and they do not always yield satisfying results.

Therefore, the added value of this paper primarily lies in the fact that we have proposed a methodology that deals with datasets that have a small amount of data. In fact, high amounts of data are hard to obtain in the medical field due to requiring ethical approvals and the need to ensure data privacy protection. As a result, this paper focuses on the novel methods that could be used on small datasets and can thus surpass traditional data enhancement methods. Although applied on a specific dataset regarding the assessment of the risk of suffering an acute myocardial infarction, the proposed methodology can be translated to other medical datasets as well. In addition, the novelty of the paper lies in the validation of the proposed methodology with simulated FFRs via the finite element method (FEM). The proposed approach would reduce the time needed for diagnosis and works to eliminate invasive coronography, as the data used in this paper were faster and easier to obtain than the real measurements of FFR.

In future research, numerical calculations combined with real measurements of FFR could be used to significantly increase the size of the dataset and achieve better accuracy in the proposed ML models. In addition to the improvement of the proposed machine learning approach to assessing the risk of AMI, an additional increase in the amount of available data would enable the transfer from machine learning algorithms to creating a specialized neural network for patient classification.

## 5. Conclusions

Cardiovascular diseases are the leading cause of death globally and a major contributor to life-altering complications such as a loss in the ability to work and physical disabilities. Acute myocardial infarction occurs due to coronary artery occlusion and the insufficient oxygenation of cardiomyocytes.

The main goal of this study was to create a decision support system that is capable of classifying patients into risk classes based on their calculated fractional flow reserve. The risk classes within the final ensemble model were defined by the observed FFR value of patients, where 0.8 was chosen as a threshold value. Patients with an FFR value higher than 0.8 were viewed as belonging to the low-risk class, while those with an FFR lower than 0.8 were considered as being in the high risk-class.

In order to classify patients, an ensemble model was constructed from multiple random forest classification models, which were all trained using different combinations of training and test data. The final classification model achieved a value of 76.21% prediction accuracy. Machine learning models that showed good prediction capabilities were incorporated into the final classification ensemble, and they achieved mean prediction accuracy values of 74.1%, 77.3%, 78.1% and 83.6%, which were tested with 5%, 10%, 15% and 20% test samples, respectively.

In conclusion, we have succeeded in creating a machine learning ensemble that is capable of classifying patients based on their risk of death via a fractional flow reserve, which greatly improves prediction capabilities over a single machine learning model, even when using a small amount of available training data. Additionally, feature importance was calculated based on the training weights of the created model, which provides a possible starting point for future research and classification accuracy improvements.

## Figures and Tables

**Table 1:** Dataset description.

Name	Type	Range
Numeric FFR	Numeric	0–1
Risk class	Binary	[0, 1]
Smoker	Binary	[0, 1]
Gender	Binary	[0, 1]
Age	Numeric	36–73
CK	Numeric	61–3353
CKMB	Numeric	11–324
AST	Numeric	12–348
LDH	Numeric	210–2557
Troponin	Numeric	0.0124–58.96
pBNP	Numeric	48.1–32,700.0
CRP	Numeric	3.3–122.6
Leukocyte	Numeric	7.1–18.61
Glucose	Numeric	4.7–16.3
Urea	Numeric	5.0–23.2
Creatinine	Numeric	59.0–1447.0
Ac. uricum	Numeric	204.0–532.0
Cholesterol	Numeric	3.33–7.4
Trig	Numeric	0.88–8.3
HDL	Numeric	0.7–2.07
LDL	Numeric	2.57–5.88
Atherosclerosis index	Numeric	3.23–6.17
Cholesterol/HDL	Numeric	3.28–9.43
LAD	Descriptive/Numeric	0–1
LCx	Descriptive/Numeric	0–1
RCA	Descriptive/Numeric	0–1

**Table 2:** Feature correlations with the FFR.

Feature	Correlation with the FFR	Feature	Correlation with the FFR
Smoker	−0.39	Urea	0.15
Gender	−0.19	Creatinine	0.11
Age	0.21	Ac. Uricum	0.40
CK	−0.21	Cholesterol	−0.02
CKMB	−0.07	Trig	0.09
AST	−0.14	HDL	0.03
HDL	−0.33	LDL	0.06
Troponin	0.25	Atherosclerosis index	0.16
pBNP	0.33	Cholesterol/HDL	0.02
CRP	−0.18	LAD	0.50
Leukocyte	−0.23	LCx	0.25
Glucose	0.30	RCA	0.30

**Table 3:** Classification accuracy metrics.

Train: Test Split	Mean Prediction Accuracy
95%: 5% split	74.1%
90%: 10% split	77.3%
85%: 15% split	78.1%
80%: 20% split	83.6%
Final model	76.21%

**Table 4:** The simulated feature importance of the final model.

Feature	Feature Importance	Feature	Feature Importance
Smoker	0.023	Glucose	0.058
Gender	0.014	Urea	0.048
Age	0.048	Creatinine	0.047
CK	0.042	Ac. Uricum	0.077
CKMB	0.045	Cholesterol	0.048
AST	0.047	Trig	0.037
LDH	0.048	HDL	0.043
Troponin	0.057	LDL	0.045
pBNP	0.074	Atherosclerosis index	0.009
CRP	0.041	Cholesterol/HDL	0.059
Leukocyte	0.058	Observed coronary artery	0.032

## Data Availability

The data presented in this study are available on request from the corresponding author. The data are not publicly available due to patient privacy protection and ethical guidelines.
